# Dual addressing of thymidine synthesis pathways for effective targeting of proliferating melanoma

**DOI:** 10.1002/cam4.1113

**Published:** 2017-06-13

**Authors:** Tara Miran, Andreas T. J. Vogg, Laila El Moussaoui, Hans‐Jürgen Kaiser, Natascha Drude, Verena von Felbert, Felix M. Mottaghy, Agnieszka Morgenroth

**Affiliations:** ^1^ Department of Nuclear Medicine University Hospital Aachen RWTH Aachen University 30 Pauwelsstrasse Aachen 52074 Germany; ^2^ Department of Dermatology and Allergology University Hospital RWTH Aachen 30 Pauwelsstrasse Aachen 52074 Germany; ^3^ Department of Nuclear Medicine Maastricht University Medical Center Maastricht the Netherlands

**Keywords:** Auger electron emitter, endogenous radiation, malignant melanoma, nucleoside synthesis pathway, thymidylate synthase

## Abstract

Here, we examined the potential of blocking the thymidine de novo synthesis pathways for sensitizing melanoma cells to the nucleoside salvage pathway targeting endogenous DNA irradiation. Expression of key nucleotide synthesis and proliferation enzymes thymidylate synthase (TS) and thymidine kinase 1 (TK1) was evaluated in differentiated (MITF ^high^ [microphthalmia‐associated transcription factor] IGR1) and invasive (MITF ^medium^ IGR37) melanoma cells. For inhibition of de novo pathways cells were incubated either with an irreversible TS inhibitor 5‐fluoro‐2′‐deoxyuridine (FdUrd) or with a competitive dihydrofolate‐reductase (DHFR) inhibitor methotrexate (MTX). Salvage pathway was addressed by irradiation‐emitting thymidine analog [^123/125^I]‐5‐iodo‐4′‐thio‐2′‐deoxyuridine (^123/125^I‐ITdU). The in vivo targeting efficiency was visualized by single‐photon emission computed tomography. Pretreatment with FdUrd strongly increased the cellular uptake and the DNA incorporation of ^125^I‐ITdU into the mitotically active IGR37 cells. This effect was less pronounced in the differentiated IGR1 cells. In vivo, inhibition of TS led to a high and preferential accumulation of ^123^I‐ITdU in tumor tissue. This preclinical study presents profound rationale for development of therapeutic approach by highly efficient and selective radioactive targeting one of the crucial salvage pathways in melanomas.

## Introduction

Malignant melanoma (MM) is the fifth common cancer with dramatically increasing incidence rates 1, 2. The frequent tendency to multifocal metastatic growth causes poor prognosis, with former 5‐year survival rates of <5% 3. Based on the concept of oncogene addiction different signaling pathways have been defined as attractive therapy targets 4. Small molecular inhibitors of the MAPK pathway and of MAPK‐independent pathways have been developed and clinically evaluated for therapy of melanoma 5. Despite initial success the clinical responses are transient, and the patient relapse due to acquired resistance. As the monotherapies have not provided complete and durable responses, a dual targeting approach of crucial replenishing pathways promises a significant improvement of therapy outcome.

Here, we evaluate a novel strategy of cotargeting both the de novo and salvage thymidine synthesis pathways which are essential for increased mitotic activity, by means of chemotherapy and endogenous irradiation, respectively. Thymidylate synthase (TS) represents an attractive target for addressing the de novo thymidine synthesis in tumor cells 6. 5‐fluoro‐2‐deoxyuridine (FdUrd), the precursor of biologic active anabolite 5‐FdUrd monophosphate, is commonly used as potent and selective inhibitor of TS. In vitro FdUrd depletes intracellular thymidine triphosphate pool (TTP) and synchronizes tumor cells in the S‐phase 7, 8. In vivo, the application of FdUrd promotes the therapeutic efficiency of radiolabeled thymidine analogs in the tumor 9 as de novo pathway is blocked and the tumor cells become addicted to the salvage pathway. The use of nucleotide derivatives as a vehicle of lethal radionuclides represents an attractive Endo‐Radiotherapy (ERT) approach in the treatment of advanced neoplastic diseases 10. The particularity of this concept arises from two key aspects, the preferential and efficient incorporation of the thymidine analog ^123,125^I‐ITdU into the DNA of proliferating tumor cells and the unique radiation characteristics of the delivered Auger electron emitters like ^123,125^I 9. The action of Auger emitters is described as “single cell kill” owing to the generation of very short‐ranged (~10 nm) high ionization density clusters proximate to the DNA double strand 11. Importantly, the lethal Auger emission efficiency drops to about 1% when the emitter is located within the cytosol or on the cell membrane.

In this work, the cotargeting of crucial thymidine synthesis pathways led to highly selective and efficient incorporation of ^123/125^I‐ITdU into melanoma cells. This approach has the potential as an innovative therapeutic option for invasive mitotically active malignant melanomas.

## Materials and Methods

### Chemicals

Chemicals and solvents were purchased from Sigma‐Aldrich (St. Louis, MO) and Merck (Germany) or otherwise as indicated. All reagents and solvents were of the highest commercially available purity grade. No‐carrier‐added (n.c.a.) sodium [^125^I]‐iodide was obtained from PerkinElmer international, [^123^I]‐iodide was from GE Healthcare international. The precursor of ^123,125^I‐ITdU, precursor 5‐(trimethylstannyl)‐4′‐thio‐2′‐deoxyuridine, CAS no. 444586‐71‐4, and the unlabeled reference standard, 5‐iodo‐4′‐thio‐2′‐deoxyuridine (ITdU) CAS no. 134699‐95‐9, were synthesized as previously reported 12. Used water and acetonitrile for reagents were from Merck. ^18^F‐FLT was purchased from Max Planck Institute for Neurological Research (Cologne, Germany). Chloramine T (sodium N‐chloro‐p‐toluenesulfonamide trihydrate, CAS no. 7080‐50‐4) was from Sigma‐Aldrich. For concentration of the radionucleosides, a SPE cartridge (Sep‐Pak C18 Plus Short Cartridge, 360 mg sorbent, 55−105 *μ*m particle size, part no. WAT020515, Waters, MA) was applied, conditioned by 2 mL EtOH, followed by flushing with 15 mL water. For analytical and semipreparative chromatography the following column was used: MultoKrom 100–5 C4 (250 × 4 mm, reversed‐phase column, CS‐Chromatography, Langerwehe, Germany).

### Radiochemistry

Until the first purification via HPLC production of n.c.a. ^125^I‐ITdU and ^123^I‐ITdU were performed according to the same procedure as outlined in the following. Sixteen microliter Chloramine T (2.4 mmol/L in H_2_O: CH3CN = 2:1) was added to a mixture of 17 *μ*L phosphate buffer (0.2 mol/L, pH 2.0, in H_2_O: methanol = 7:3), 3 *μ*L precursor solution (123 mmol/L in H_2_O: methanol = 1:2), and 10 *μ*L n.c.a. ^123,125^I‐NaI solution in 0.05 mol/L NaOH. Labeling reaction completed within 10 min at RT and was stopped by addition of 10 *μ*L 100 mmol/L sodium thiosulfate. This mixture was transferred to the HPLC port and reactor and injection syringe were finally flushed with 30 *μ*L 10% EtOH aq; thus, an entire volume of 86 *μ*L was injected. The product was purified by HPLC with UV at 254 nm and gamma detection (NaI detector, 13–1600 keV) using the MultoKrom HPLC columneluted with 15% ethanol aq at a flow rate of 1 mL/min. Retention times were as follows: 3.4 min for ^123,125^I‐iodide and 6.3 min for ^123,125^I‐ITdU. Product volume was 1.0 ± 0.2 mL. Quality control was performed by a method identical to that of the HPLC separation. Total radiochemical yields were 80 ± 8%. Radiochemical purities were >97%. Molar concentrations of prepared n.c.a. ^125^I‐ITdU solutions in 15% ethanol aq. were 1.3 *μ*mol/L (±30%) with typical activity concentrations of 50 MBq/mL (with batch variations ± 20 MBq/mL) and corresponding specific activities of 40 GBq/*μ*mol. Analogous n.c.a. ^123^I‐ITdU solutions achieved concentrations of 0.3 *μ*mol/L (±30%) with typical activity concentrations of 130 MBq/mL (with batch variations ± 100 MBq/mL) and corresponding specific activities of 660 GBq/*μ*mol. These radionucleoside solutions were ready for cell uptake experiments. However, for application to mice the following concentration procedure became mandatory. Ca. 1.5 mL ^123^I‐ITdU solution was diluted by 18 mL of water plus 0.1 mL 50% HOAc aq and passed through the preconditioned Sep‐Pak C18 Plus Short Cartridge, blow‐dried with argon, rinsed with additional 1 mL of water, followed by a final blow drying with argon. The trapped nucleoside was eluted by MeCN. The initial 0.4 mL of effluent was discarded and the subsequent 0.8 mL was collected in a v‐shaped vial and evaporated in an argon stream at 85°C. After ca. 10 min additional 200 *μ*L MeCN was added and the solution was evaporated just to dryness. For formulation 60 *μ*L EtOH and 600 *μ*L PBS were added. This final solution (100 *μ*L, diluted with further 100 *μ*L PBS) was used for animal application. Final quality control using the above‐mentioned method proved radiochemical purities of >97%. Overall radiochemical yields were 51 ± 5 MBq.

### Cell lines and culture conditions

Human malign melanoma cell lines IGR1 and IGR37 were obtained from DSMZ (Braunschweig, Germany). All cells were cultured in 85% Dulbecco's MEM supplemented with 15% h.i. FBS (Biochrom, Berlin, Germany). The cell lines were authenticated by the STR profiling. All the cell cultures were tested for Mycoplasma contamination by PCR before use. For stimulation, the cells were incubated for 24 h, with 5‐fluoro‐2‐deoxyuridine (FdUrd, 10 *μ*mol/L) or with methotrexate (MTX, 10 *μ*mol/L).

### Flow cytometry and immunocytochemistry

The proliferation status was determined using a BrdU and 7‐AAD (7‐amino‐actinomycin D, total DNA) staining (BD Pharmingen, San Diego, CA) according to the manual instruction. The stained cells were analyzed by flow cytometry (FACS, Cytomics FC 500, Beckman Coulter, Germany). The FACS data were analyzed using CXP Software (Beckman Coulter). For bright‐field microscopy analysis the cells were plated onto poly‐lysine‐coated coverslips and fixed with 4% PFA. Nuclei were counterstained using Hoechst33342 (1 mg/mL, Sigma‐Aldrich), before being examined by fluorescence microscopy (Axio Scope A1, ZEISS, Germany).

### Staining with *γ*H2AX

MTX or FdUrd (10 *μ*mol/L each for 24 h) stimulated IGR37 cells were treated with ^125^I‐ITdU (200 kBq for 24 h). After wash steps with PBS the cells were fixed with 4% PFA and permeabilized by ice cold methanol (100% for 10 min −20°C). After wash steps with PBS the cells were blocked with 5% BSA and 0.3% Triton X‐100 for 60 min at RT. The cells were incubated at 4°C overnight with the primary *γ*H2AX pSer^139^‐specific antibody (1:800 diluted in 1% BSA and 0.3% Triton X‐100, Cell Signaling, Danvers). After wash steps with PBS, the cells were incubated for 1 h at RT with antirabbit AlexaFluor555‐conjugated antibody (1:500 in 1% BSA and 0.3% Triton X‐100, Cell Signaling). The cells were washed with PBS and analyzed by flow cytometry. For fluorescence microscopy (Axio Scope A1), the cells were additionally counterstained with Hoechst33342 (1 mg/mL, Sigma‐Aldrich).

### Evaluation of intracellular GSH concentration by ELISA

The intracellular GSH level was assessed by using Glutathione Fluorometric Assay Kit (BioVision, San Francisco). The treated cell (2*10^6 per sample) was homogenized on ice with 100 *μ*L of ice cold Glutathione Assay Buffer. To 60 *μ*L of the cell homogenate was added 20 *μ*L perchloric acid (6N) and further homogenized by vortexing for several seconds. After incubation on ice (5 min) the samples were centrifuged for 2 min at 13,000*g* and 4°C. The collected supernatant (40 *μ*L) was neutralized with 20 *μ*L ice cold KOH and incubation for 5 min on ice. After centrifugation for 2 min at 13,000*g*, 4°C samples (10 *μ*L) were added to 90 *μ*L of assay buffer. The samples were read on a fluorescence plate reader (Infinite F200, Tecan, Switzerland) after incubation with o‐phthalaldehyde (10 *μ*L) for 40 min at RT. The glutathione concentration was determined based on the standard curve. All samples were performed in triplicates.

### Cellular uptake and DNA incorporation of ^125^I‐ITdU

For uptake experiments, the untreated and pretreated cells (1*10^4^/well) were washed with PBS and cultured in medium supplemented with 200 kBq of ^125^I‐ITdU. After 1, 4, and 24 h, the cells were washed three times with PBS and the intracellular accumulated radioactivity was assessed by a gamma counter (Wizard2, PerkinElmer, Waltham, MA). After measurement, DNA was extracted using the DNeasy Tissue Kit (Qiagen, Germany). Incorporated radioactivity was measured in a gamma counter. The experiments were carried out three times in triplicates.

### Tumor model

A suspension of 1*10^6 IGR37 cells was injected subcutaneously into the neck of 6‐ to 8‐week‐old female NOD SCID mice (Charles River, Wilmington, Massachusetts). After 4 weeks, the mice developed subcutaneous tumors of about 100 mm diameter. All animal procedures and experiments were performed in accordance with the guidelines of the German Regulations of Animal Welfare. The protocols were approved by the local Ethical Committee for Animal Experiments.

### Biodistribution study

Thirty minutes before intravenous injection of ^18^F‐FLT (5 MBq in 50 *μ*L saline) or ^123^I‐ITdU (10 MBq in 100 *μ*L saline), FdUrd (5 mg/kg in 50 *μ*L saline) was injected into the lateral tail vein of NOD SCID mice bearing xenotransplanted IGR‐37 tumors. The biodistribution of ^18^F‐FLT was evaluated 60 min p.i. by using the microPET (Inveon, Simens, Knoxville). CT images were produced by a Philips Gemini TF16 PET/CT (Philips Medical Systems, Best, The Netherlands). The PET images were reconstructed using the iterative OSEM3D/MAP (OSEM3D 2 iterations, MAP 18 iterations) algorithm. The biodistribution of ^123^I‐ITdU was evaluated 4 and 24 h p.i. by using a clinical dual‐head SPECT Siemens E.cam gamma camera equipped with *μ*SPECT pinhole collimators. Subsequent CT images were produced by a Philips Gemini TF 16 PET/CT. The corresponding SPECT images were reconstructed using an iterative OSEM (5 iterations, 16 subsets) algorithm representing activity concentrations in units of kBq/cc. Calculation of activity uptake values (kBq) was performed by VOI definition in tumor, liver, and spleen as well as percentage uptake related to the injected activity.

### SDS/Western blot analysis

For SDS‐PAGE/Western blot analysis, the protein lysates were prepared by lysis with Tris‐HCl buffer, NP‐40 (1%), PMSF (1 mmol/L), and inhibitor cocktail (Roche, Switzerland). Protein samples were separated by 4–20% SDS‐PAGE (BioRad, Berkeley, CA) and transferred onto PVDF membrane. Protein detection was performed with polyclonal antibodies: anti‐DNA PKC (1:200, abcam), anti‐ChK2 (1:1000, Cell Signaling), anti‐phospho ChK2 (1:1000, Cell Signaling), anti‐ATM (1:1000, abcam, Cambridge, UK), anti‐phospho ATM (1:1000, abcam), anti‐TS (1:200, abcam), anti‐ MITF (1:1000, abcam), anti‐TK1 (1:10000, abcam), anti‐Caspase3 (1:1000, Cell Signaling), anti‐cleaved Caspase3 (1:1000, Cell Signaling), anti‐PARP (1:1000, Cell Signaling), and anti‐cleaved PARP (1:1000, Cell Signaling). The secondary IgG coupled to HRP (1:2000, Cell Signaling) was visualized with enhanced chemiluminescence (ECL+, GE Healthcare, UK). Equal protein loading was controlled using GAPDH‐specific antibody (Ambion, Life Technologies, Carlsbad, CA) and secondary goat antimouse IgG linked to HRP (abcam). The experiments were carried out three times in triplicates. The bands were detected using the ImageQuant LAS 4010 camera system (GE Healthcare).

### DNA extraction from tumor, spleen, and liver

The DNA isolation was accomplished by using DNeasy Blood and Tissue Kit (Qiagen, Venlo, Netherlands) according to the manufacturer's instructions. Four‐hour and 24‐h after injection of ^123^I‐ITdU, tumor, spleen, and liver tissues were dissected and incubated at 56°C for 3 h in tissue lysis buffer and proteinase K. After washing and elution steps, the amount of isolated DNA was determined. The radioactivity in DNA and in other tissue fractions was analyzed with a gamma counter and results were given in cpm/*μ*g isolated DNA.

### In situ nick end labeling assay

Consecutive formalin‐fixed, paraffin‐embedded tissue sections (2 *μ*m) were dewaxed in xylene and rehydrated through graded concentrations of ethanol to distilled water. Terminal deoxynucleotidyl transferase‐mediated dUTP nick end labeling assay was done according to the manufacturer's instructions (Roche, Basel, Switzerland). Color development used diaminobenzidine substrate and sections were counterstained with hemalaun. The cellular apoptotic effects were examined using a Zeiss microscope.

### Immunohistochemistry of tumor tissue sections

Consecutive formalin‐fixed, paraffin‐embedded tissue sections (2 *μ*m) were dewaxed in xylene and rehydrated through graded concentrations of ethanol to distilled water. Sections were then immersed in 10 mmol/L citrate buffer (pH 6.0) and processed in thermostatic water bath for 30 min at 98°C for antigen retrieval. After the antigen retrieval treatment, the tissue sections were incubated for 60 min with Ki67 (1:100), anti‐TS (1:100), anti TK1 (1:100), and anti‐MITF (1:250) antibodies. Subsequently, the sections were exposed for 60 min to peroxidase‐linked antimouse immunoglobulin antibody (polymer from DAKO, Santa Clara, CA) or peroxidase‐linked donkey antirat immunoglobulin antibody (dilution 1:250; Dianova, Torres Vedras, Portugal). Color development used diaminobenzidine substrate and sections were counterstained with hemalaun.

### Statistical analysis

Data are presented as mean ± standard deviation. All statistical calculation were performed using Graph Pad Prism version 6.00 (San Diego California) for Windows. Data were analyzed by one‐way and two‐way ANOVA with Post hoc comparisons were performed with Sidak's, Bunnett's, and Tuckey's multiple comparison tests. Effects were considered to be statistically significant if *P* ≤ 0.05.

## Results

### Targeting of the de novo pyrimidine synthesis pathway promotes proliferation in melanoma

**Figure 1:**
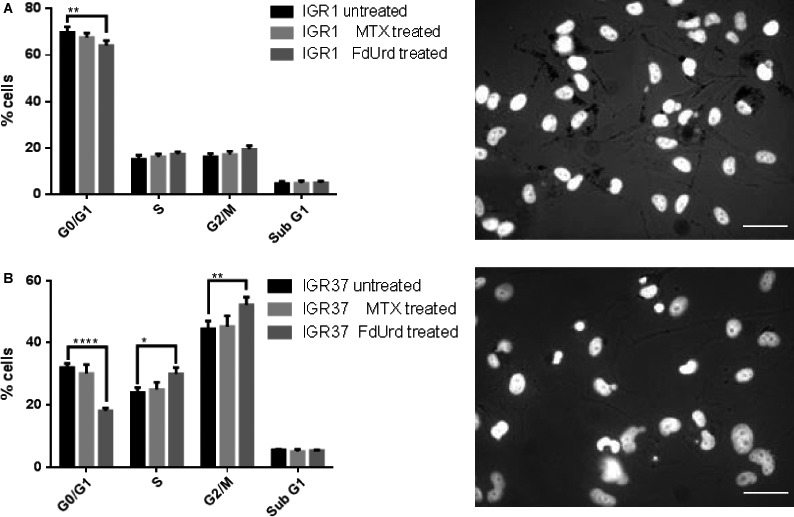
Flow cytometric cell cycle analysis *(left)* and microscopic phenotype analysis of IGR1 and IGR37 cells *(right)*. Histogram data of BrdU and 7‐AAD stained untreated, MTX (10 *μ*mol/L, 24 h), or FdUrd (10 *μ*mol/L, 24 h) treated IGR1 cells (A) and IGR37 cells (B). Bright‐field microscopy visualizes pigmentation (black grains) of untreated IGR1 and IGR37 cells. DNA was counterstained with Hoechst33342. Scale bar = 50 *μ*m. Data represent means ± SD from three experiments. *P < 0.05 by two‐way ANOVA with Tuckey's multiple comparisons test.

To evaluate the effects of TS inhibition melanoma cell lines IGR1 and IGR37 were incubated for 24 h with two different de novo thymidine synthesis pathways targeting drugs MTX (10 *μ*mol/L) and FdUrd (10 *μ*mol/L). The IGR1 cells with low mitotic index and intense pigmentation represent the differentiated melanoma phenotype, while the mitotically active and only mild pigmented IGR37 display the proliferative melanoma status. After treatment with MTX, an inhibitor of DHFR, no effects on mitotic activities were detected in both cell lines (Fig. 1). In contrast, FdUrd highly increased the proliferation of IGR37, but failed to impact the mitotic activity in differentiated IGR1 cells. Importantly, none of these inhibitors affected the cell viability.

### Targeting of the de novo nucleoside synthesis activates the checkpoint signaling pathways in mitotically active melanoma

**Figure 2:**
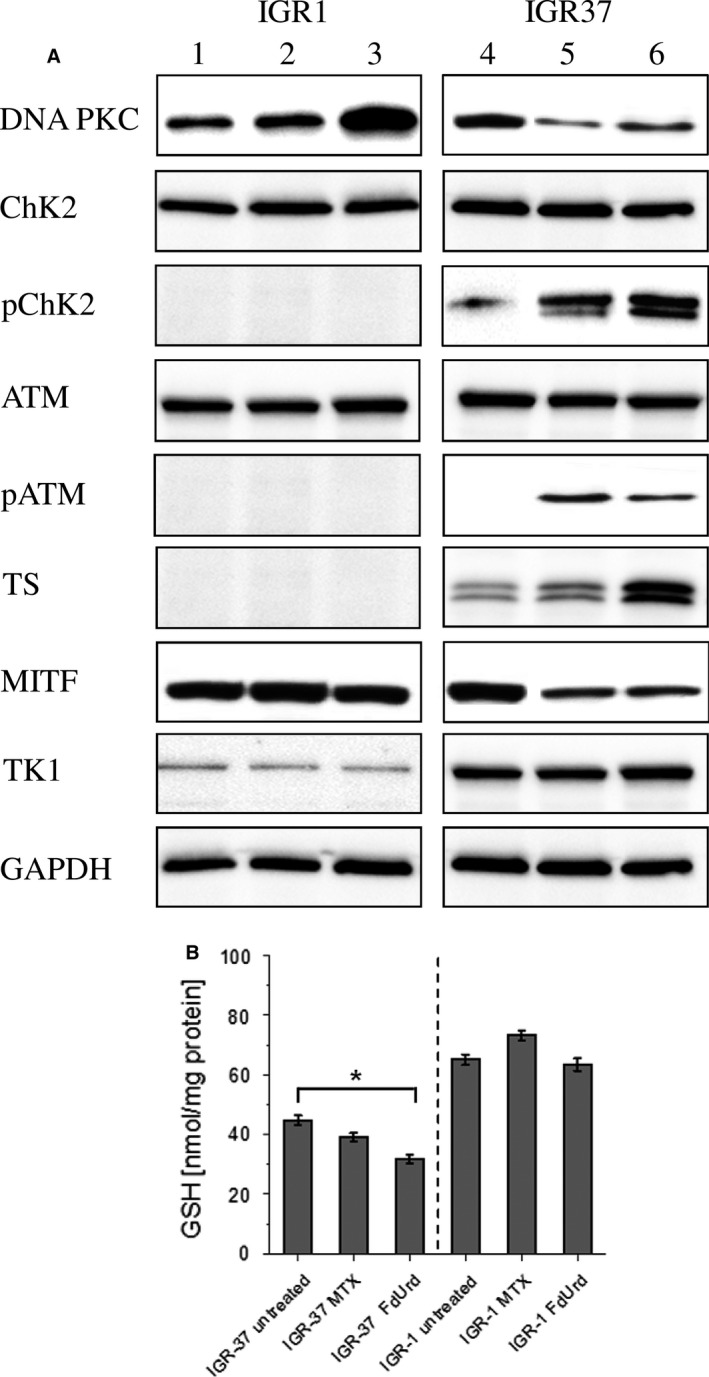
Analysis of MTX and FdUrd induced effects on prosurvival pathways in IGR1 and IGR37 cells. SDS‐PAGE and Western Blot analysis of activation of checkpoint response, DNA repair mechanisms, and expression of proteins involved in de novo and salvage nucleoside synthesis pathways in response to MTX (10 *μ*mol/L, 24 h, lane 2, 5) and FdUrd (10 *μ*mol/L, 24 h, lane 3, 6) in IGR1 and IGR37 cells. Lanes 1 and 4 represent untreated IGR1 and IGR37 cells, respectively. GAPDH served as a loading control (A). Enzyme‐linked immunosorbent assay of GSH concentration in IGR1 and IGR37 cells after treatment with MTX (10 *μ*mol/L, 24 h) and FdUrd (10 *μ*mol/L, 24 h). Data represent means ± SD from three experiments. *P < 0.05 by one‐way ANOVA with Dunnett's multiple comparisons test (B).

To assess the molecular effects of MTX and FdUrd on the relevant checkpoint and repair signaling pathways, we compared their ability to impact the expression and activation of DNA PKC, ATM, and ChK2 (Fig. 2A). The results indicate that both drugs induced the phosphorylation of ATM and ChK2 solely in IGR37 cells. Simultaneously, FdUrd but not MTX strongly increased the expression of DNA PKC, the key activator of nonhomologous end‐joining (NHEJ) pathway of DNA repair in IGR1 cells 13. By contrast, both drugs decreased the DNA PKC expression in the proliferating IGR37 cells. Moreover, because MITF acts as an essential regulator of melanoma cell viability and survival 14, the reduced expression of DNA PKC in IGR37 cells was accompanied by a decline in the MITF expression. The poor susceptibility of IGR1 cells to the drugs may partially be explained by virtually no expression of one of the targeted enzymes, the TS. The low expression of TK1 in IGR1 cells may additionally limit the FdUrd potential, as TK1 catalyzes the necessary conversion of FdUrd to FdUMP, the actual inhibitor of TS 15. Treatment with FdUrd but not with MTX affected the major antioxidant defense mechanism (Fig. 2B). Namely, the inhibition of the de novo pyrimidine synthesis significantly reduced the intracellular concentration of glutathione (GSH) in proliferating IGR37 cells. By contrast, in IGR1 cells MTX led to an increase in the GSH concentration while FdUrd failed to impact significantly the intracellular redox potential.

### FdUrd efficiently increases the cellular uptake and DNA incorporation rate of thymidine analogs

**Figure 3:**
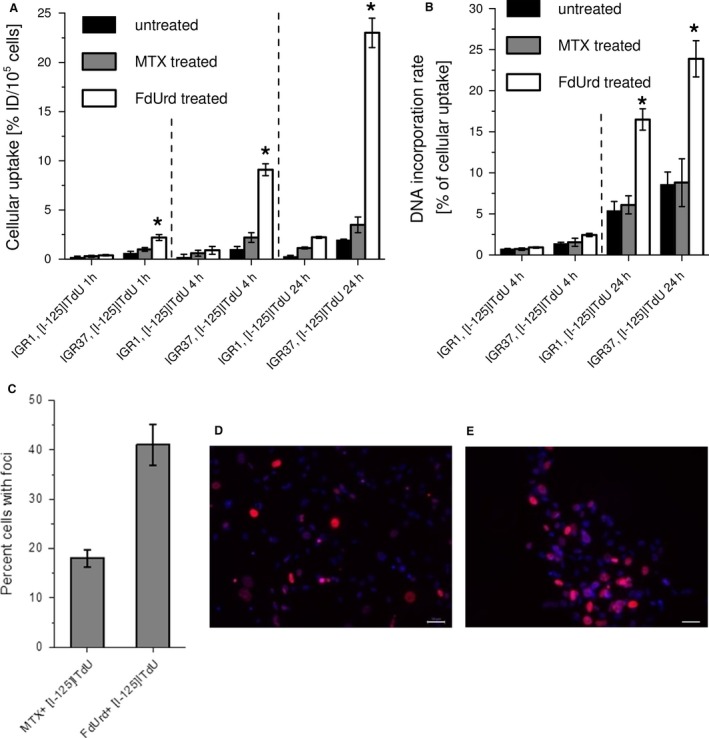
Effect of MTX and FdUrd on cellular uptake and incorporation into the DNA of ^125^I‐ITdU in IGR1 and IGR37 cells. Uptake (%ID/well) of ^125^I‐ITdU (200 kBq/well, 1, 4, and 24 h) in IGR1 and IGR37 cells in dependency on MTX or FdUrd (both 10 *μ*mol/L, 24 h) stimulation. (A). DNA incorporation of ^125^I‐ITdU (200 kBq/well, 4 and 24 h) into IGR1 and IGR37 cells in dependency on MTX or FdUrd (both 10 *μ*mol/L, 24 h) stimulation. Data represent means ± SD from three experiments. *P < 0.05 by one‐way ANOVA with Sidak's multiple comparisons test (B). FACS quantification (*P < 0.05 by one‐way ANOVA with Dunnett's multiple comparisons test) of *γ*H2AX foci in IGR37 cells after treatment with MTX and ^125^I‐ITdU (200 kBq, 24 h) or with FdUrd and ^125^I‐ITdU (200 kBq, 24 h) (C). Corresponding fluorescence microscopy of *γ*H2AX foci in IGR37 cells after treatment with MTX and ^125^I‐ITdU (200 kBq, 24 h) (D), and after treatment with FdUrd and ^125^I‐ITdU (200 kBq, 24 h) (E). DNA was counterstained with Hoechst33342. Scale bar = 50 *μ*m.

To examine the MTX‐ or FdUrd‐mediated effect on the efficiency of the salvage nucleoside synthesis pathway addressing thymidine analog ^125^I‐ITdU, both melanoma cell lines were investigated regarding the cellular uptake and DNA incorporation of ^125^I‐ITdU after 1, 4, and 24 h (Fig. 3A and B). Generally, in accordance with the low mitotic activity reflected by low TK1 expression, the IGR1 cells showed only minor uptake rate of ^125^I‐ITdU. The time‐dependent increase in ^125^I‐ITdU uptake and intracellular accumulation was most pronounced in FdUrd‐treated IGR1 cells. By contrast, consistent with the stimulation study, the capacity of FdUrd to modulate proliferation of IGR37 cells resulted in strong increase in ^125^I‐ITdU uptake. Similar to IGR1 cells, in IGR37 cells MTX affected the ^125^I‐ITdU uptake to a considerably lower extend than FdUrd. After cellular uptake, ^125^I‐ITdU was shown to follow the metabolic pathway of exogenously supplied thymidine, the nucleoside salvage pathway 8, 16. The incorporation into DNA is crucial for efficient targeting of malignant cells by molecules bearing Auger electron emitters 11. Corresponding to the low efficiency of ITdU uptake, the IGR1 cells only marginally incorporated the thymidine analog into the DNA irrespective of the applied stimulation. Hence, <2.5% of the intracellularly accumulated tracer was used as a substrate for DNA polymerase. In contrast, IGR37 cells exhibited higher incorporation rates of ^125^I‐ITdU into the DNA even without preconditioning. While MTX failed to impact on the DNA incorporation step, FdUrd significantly increased mitotic activity which resulted in an elevated incorporation of ^125^I‐ITdU into the DNA.

### FdUrd‐mediated inhibition of the de novo pyrimidine synthesis pathway renders proliferating melanoma cells sensitive to endogenous radiation

The results so far indicated that solely the proliferating stadium of melanoma cells responds to the strategy of simultaneously targeting thymidine synthesis pathways. Consistent with the effects on the cellular uptake and, more importantly, on the incorporation of ^125^I‐ITdU into the DNA in IGR37 melanoma cells, flow cytometric and microscopy analyses revealed that addressing de novo pyrimidine synthesis pathway by FdUrd but not by MTX led to an significantly increased accumulation of *γ*H2AX foci which visualize the DNA double‐strand breaks (DSBs) in the DNA after incubation with 200 kBq ^125^I‐ITdU for 24 h (Fig. 3C).

### Inhibition of the de novo thymidine synthesis pathway leads to a preferential accumulation of ^123^I‐ITdU in tumor tissue

**Figure 4:**
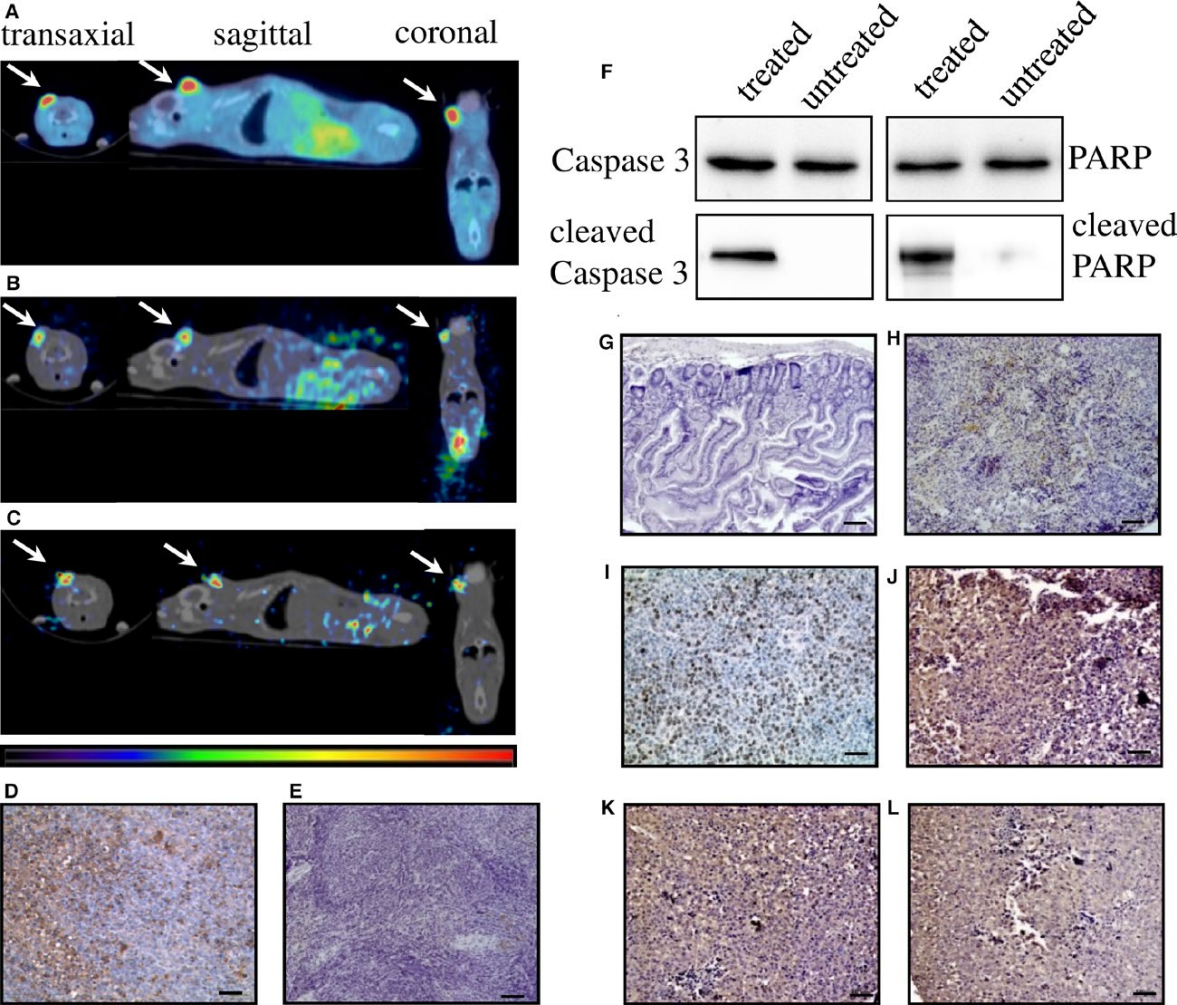
Molecular imaging of^18^F‐FLT and ^123^I‐ITdU biodistribution in IGR37‐xenografted mice model and ex vivo histological staining analyses. *μ*PET/CT biodistribution analysis 1 h p.i. of ^18^F‐FLT (5 MBq) in FdUrd pretreated mice (5 mg/kg 30 min prior to ^18^F‐FLT application) (A). *μ*SPET/CT biodistribution analysis 4 h p.i. of ^123^I‐ITdU (10 MBq, in FdUrd pretreated mice (5 mg/kg 30 min prior to ^123^I‐ITdU application) (B). *μ*SPET/CT biodistribution analysis 24 h p.i. of ^123^I‐ITdU (10 MBq) in FdUrd pretreated mice (5 mg/kg 30 min prior to ^123^I‐ITdU application) (C). Arrows indicate tumor localization. (*n* = 5) TUNEL staining in tumor sections extracted from mice (48 h p.i.) treated with FdUrd (5 mg/kg) and ^123^I‐ITdU (10 MBq) (D) and in tumor sections extracted from untreated mice (E). Immunoblot analysis of activation of intrinsic apoptotic pathway in tumor extracted from mice (48 h p.i.) treated with FdUrd (5 mg/kg) and ^123^I‐ITdU (10 MBq) and from untreated animals (F). TUNEL staining in small intestine (G) and spleen (H) extracted from mice (48 h p.i.) treated with FdUrd (5 mg/kg) and ^123^I‐ITdU (10 MBq). Detection of Ki67 (I), MITF (J), TS (K), and TK1 expression (L) in tumor extracted (48 h p.i.) from mice treated with FdUrd (5 mg/kg) and ^123^I‐ITdU (10 MBq). The tissue sections were stained with HE. Scale bar = 50 *μ*m. (*n* = 5).

To investigate the potential of FdUrd‐mediated inhibition for an increase in salvage pathway‐mediated thymidine supply in vivo, we visualized the biodistribution of ^123^I‐ITdU (10 MBq) after pretreatment with FdUrd in IGR37 xenografted mice (Fig. 4A–C, Table 1). The SPECT acquisitions performed at 4 and 24 h p.i. imaged the uptake and retention of ^123^I‐ITdU in both tumor and normal tissues. The pretreatment with FdUrd (5 mg/kg, 30 min prior to ^123^I‐ITdU) led to a highest radioactivity uptake in the melanoma xenograft compared to tissue with physiological high proliferation rates like spleen and to tissue with low proliferation rate like the liver. The initial high concentration in spleen is probably due to the transient uptake of ITdU into proliferating cells and cells undergoing apoptosis, as the red pulp of the spleen in rodents is the site of extramedullary hematopoiesis and degradation of dead cells 17. The detected radioactivity in the bladder reflects the renal elimination of the nonincorporated ^123^I‐ITdU and the free radioiodine generated by deiodination. Importantly, at 24 h p.i. the highest retention of ^123^I‐ITdU was detected in the tumor tissue while the concentration of radioactivity in normal tissues diminished progressively. A parallel *μ*PET analysis with the standard nucleoside PET tracer ^18^F‐FLT revealed a similar uptake pattern thus confirming the highly selective uptake and retention of ^123^I‐ITdU in the tumor tissue. Correspondingly, the investigation of the ^123^I‐ITdU distribution among cell compartments of retaining tissue revealed the highest incorporation rate of ^123^I‐ITdU in the DNA of malignant cells (Table 1). To determine the radiotoxic potential of our cotargeting strategy, we assessed the extent of apoptosis 48 h after ^123^I‐ITdU application. As a result the tumor tissue resected from treated mice showed a high rate of apoptosis, while no apoptosis was visible in control animals (Fig. 4D and E). Correspondingly, activation of the intrinsic apoptotic pathway was exclusively detected in FdUrd‐ and ^123^I‐ITdU‐treated animals (Fig. 4F). Importantly, the viability of radiosensitive tissues like small intestine and spleen remained unaffected (Fig. 4G and H). Ki‐67 and MITF staining analysis of corresponding tissue sections showed a clear correlation between cellular ITdU uptake and the proliferation signature of the melanoma cells (Fig. 4I and J). The TS and TK1 staining proved the pronounced enzyme expression in the IGR37 xenograft tumor tissue (Fig. 4K and L).

**Table 1:** Comparison of tissue uptake and DNA incorporation rate of ^123^I‐ITdU in DNA of tumor, spleen, and liver

Tissue uptake of ^123^I‐ITdU [% of injected activity]		
In tumor	In spleen	In liver
2.01 ± 0.20% (4 h)	0.77 ± 0.08% (4 h)	0.61 ± 0.05% (4 h)
1.47 ± 0.10% (24 h)	0.20 ± 0.03% (24 h)	0.07 ± 0.01% (24 h)
Radioactive count rate of ^123^I‐ITdU per *μ*g DNA [cpm]		
168.5 ± 38.1 (24 h)	18.2 ± 2.3 (24 h)	2.1 ± 0.3 (24 h)

Data were collected post mortem from sampled tissue and represent means ± SD from three experiments.

## Discussion

**Figure 5:**
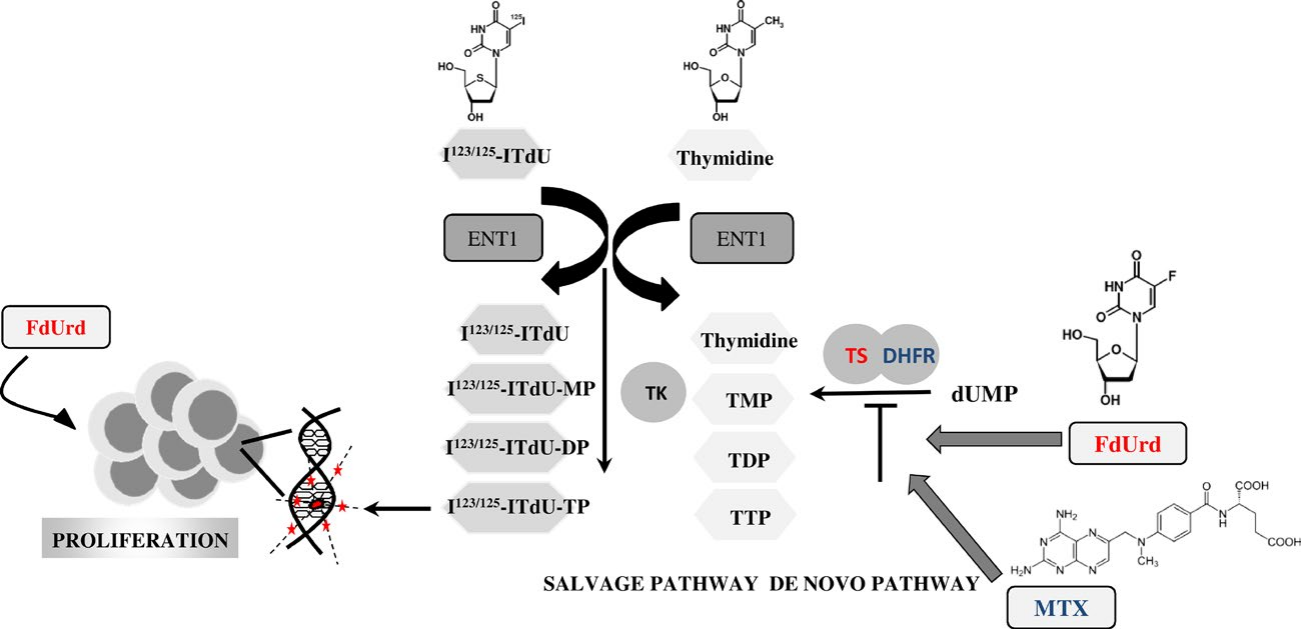
Summary of FdUrd/^123/125^I‐ITdU therapeutic strategy. FdUrd increases mitotic activity in melanoma cells and inhibits pyrimidine de novo synthesis pathway, leading to efficient uptake of salvage thymidine synthesis pathway targeting Auger electron‐emitting thymidine analog ^123/125^I‐ITdU. The DNA‐incorporated ^123/125^I‐ITdU induces severe DNA damages (double‐strand breaks) and consequently cell apoptosis.

The proliferation status was shown to be a common denominator in both disease stages, primary, and metastatic melanoma 18. Thus, targeting of proliferating melanoma cells may provide an attractive therapy option. The dual pyrimidine synthesis pathways addressing strategy used here (Fig. 5) is highly effective and selective in vitro and in vivo. First, by inhibition of TS, FdUrd potentially stimulates the proliferation and activates the checkpoint signaling pathways in metastatic melanoma cells, which in the second step are susceptible to the subsequent targeting of the counterbalancing salvage thymidine synthesis pathway by a radiolabeled thymidine analog. The melanoma cell lines used in this study respond very differently to the two TS inhibitors MTX and FdUrd. Both cell lines originate from metastatic melanoma in lymph nodes, however, the IGR1 cells exhibit stronger pigmentation than the IGR37 cells which is due to the higher 5‐S‐cystinyldopa (5‐SCD) concentration and increased tyrosinase (Tyr) activity in IGR1 cells 19, 20, 21. Despite a roughly equal expression level of MITF, the IGR1 melanocytes are mitotically considerably less active than the IGR37 cells (Fig. 1). In melanoma, MITF was shown to be a key regulator of both cell cycle and differentiation genes 22. Considering the MITF‐induced upregulated transcription of TYR, MLANA, DCT, and TYRP1 23 and the low mitotic activity (marginal TK1 expression, Figure 1 and 2), the IGR1 cells present the well‐differentiated melanocyte signature. By contrast, the TK1‐related high mitotic activity associated with a poor pigmentation and upregulated expression of SOX‐10 and cdk2 23 reflects the proliferative melanocyte signature of IGR37 cells. Due to the marginal TS expression, the well‐differentiated IGR1 cells are insensitive to the treatment with FdUrd and MTX (Figs. 1 and 2). The treatment with MTX – an inhibitor of DHFR, an enzyme synthetizing a cofactor required for the de novo synthesis of thymidine and purines – failed to impact the proliferation of both cell lines. The different effect of MTX and FdUrd could be due to the natural resistance mechanisms to MTX described in human melanoma cells 24. The intrinsic resistance to MTX is attributed to increased intracellular levels of DHFR and to increased melanosome‐mediated drug efflux 25, 26, 27. Consequently, the intracellular MTX level appears to be too low to affect the proliferation index and the cell viability. In contrast to the effect in IGR1 cells, FdUrd significantly increased the proliferation of IGR37 cells (Fig. 1). This is partially due to the increased expression of the two proteins TK1 (Fig. 2) and the nucleoside transporter protein hENT1 28, both promoting the enhanced nucleoside consumption. Importantly, the IGR37 cells compensated the inhibition of the de novo synthesis pathway by an increased intracellular uptake of ^125^I‐ITdU followed by its incorporation into the DNA via the salvage pathway (Fig. 3). This final step presents a fundamental requirement for successful therapeutic application of Auger electron emitters 29. The failure to deplete the dTTP pool in melanoma 30 results in only marginal impact of MTX on the cellular uptake of ^125^I‐ITdU and its incorporation into the DNA. In the presented study, the DNA incorporated ^125^I‐ITdU‐induced extensive DNA double‐strand breaks predominantly in FdUrd sensitized IGR37 cells. Considering the MITF‐mediated regulation of melanoma cell survival and viability 31, a decreased expression of MITF induced by MTX and FdUrd would make the cells radiosensitive. However, only FdUrd reduced the intracellular level of the main antioxidant molecule GSH, which in turn renders the cells more susceptible to ionizing radiation. Interestingly, the translation of TS was shown to be redox sensitive 32. This explains the FdUrd‐induced changes in the cellular redox state resulting in increased synthesis of TS (Fig. 2). The elevated GSH consumption in proliferative melanoma cells may be explained by the participation of GSH in error‐free DNA replication and repair 33. Importantly, the crucial benefit of using FdUrd is not limited to the quiescence of the de novo thymidine synthesis pathway by blocking TS irreversibly, but moreover, it inhibits the TS‐mediated dehalogenation of ^125^I‐ITdU. The released [^125^I]‐iodide would strongly limit the DNA proximity restricted efficiency of the delivered Auger electron emitter 8, 34. Thus, targeting of the de novo synthesis pathway by FdUrd provides an optimal conditioning of proliferating melanoma cells for the salvage synthesis pathway exploiting ^125^I‐ITdU.

For targeted ERT of melanoma different molecules and mechanisms have been identified and evaluated in preclinical and clinical studies 35. Among them, the radiolabeled benzamide derivatives targeting melanin demonstrated promising pharmacokinetic profiles 36, 37, 38. However, variation in the pigment concentration (amelanotic and poorly melanized melanomas, amelanotic metastasis) strongly impacts the efficiency of these therapies. Thus, targeting of proliferating melanoma cells regardless their melanin content may bridge the gap in therapy options of melanomas. In our in vivo *μ*SPECT/CT biodistribution analysis in an IGR37 xenografted mouse model, ^123^I‐ITdU showed an effective and highly preferential uptake and retention in the tumor tissue (Fig. 4, Table 1). The residual low background activity mainly concentrated in stomach content results from steady systemic dehalogenation processes of ^123^I‐ITdU. This efficient and preferential incorporation of ITdU into the DNA of malignant cells, leading to a massive and selective damage of tumor tissue, indicates that the combined application of ^123^I‐ITdU und FdUrd might provide an attractive Auger radiation therapy approach. In an earlier clinically evaluated ERT with the Auger emitter ^111^In‐labeled DOTATOC – which after internalization is predominantly accumulated in the cytosol – the measured uptake of 0.1% of the injected dose was assessed to deliver an absorbed dose between 36 and 344 Gy, depending on the tumor volume (10 mL vs. 1 mL 39). In this study the measured tumor uptake was almost 15 times higher (Table 1). Therefore, we conclude that the assumed absorbed dose in the tumor will be much higher (way above 500 Gy depending on tumor size) than with established ERT. This is due to the efficient incorporation of ITdU into the DNA, where the cytotoxic effect of the Auger emitter is the highest. The bone marrow dose for a mouse was calculated based on two previous preclinical studies 9, 40 using a nucleoside analog to be 60.5 *μ*Gy/MBq. Applying the weighting W_R_ factor for human the bone marrow cells would be 605 *μ*Gy/MBq, which would allow applying up to 3.3 GBq ^123^I‐ITdU combined with FdUrd in patients resulting in an equivalent bone marrow radiation absorbed dose of just below the maximal tolerated dose of 2 Gy. The potential of FdUrd for a tumor directed effect is an implication of the unique metabolic and phenotypic signatures in tumor and normal cells. Due to the overexpressed TK1 (10‐ to 100‐fold with respect to normal proliferating cells 41 tumor cells exhibit a higher potential for conversion of nucleoside analogs into active nucleotides ITdU to ITdU‐TTP and FdUrd to the inhibitory acting FdUMP 42. Additionally, the increased activity of catabolic enzymes thymidine phosphorylase and thymidine phosphatase in normal cells led to only transient retention of intact thymidine analogs in normal tissue 43. Thus, upregulation of the thymidine salvage pathway in tumor cells simultaneously with upregulation of thymidine phosphate catabolism in normal tissue might explain preferential tumor uptake and retention of ITdU.

As FdUrd‐mediated inhibition of the de novo pathway induces the salvage pathway‐dependent proliferation, we conclude that a combined therapy with FdUrd and ^123/125^I‐ITdU provides an attractive option for a treatment of proliferating melanoma. Our in vitro and in vivo results support the feasibility and the highly selective cytotoxic targeting using this dual strategy approach.

## Conflict of Interest

None declared.
